# Affective Instability: Impact of Fluctuating Emotions on Regulation and Psychological Well-Being

**DOI:** 10.3390/bs14090783

**Published:** 2024-09-06

**Authors:** Jennifer Dork, Erin Mangan, Lawrence Burns, Eugene Dimenstein

**Affiliations:** Psychology Department, Grand Valley State University, Allendale, MI 49417, USA; blakelje@gmail.com (J.D.);

**Keywords:** affective lability, affective instability, emotion regulation, well-being

## Abstract

Previous research has focused on understanding the occurrence of intense and fluctuating emotions and the ability to manage these emotions and affective states. These phenomena have been, respectively, labeled as affective instability and emotion regulation and have been studied among individuals diagnosed with borderline personality disorder (BPD), attention-deficit/hyperactivity disorder (ADHD), bipolar disorder (BD), and post-traumatic stress disorder (PTSD). Previous findings suggest that affective instability may be associated with poorer psychological well-being. The present study aims to investigate the general tendency of affective instability and capacity for emotional regulation among college students, regardless of a previous psychological diagnosis, and to understand the relationship between these processes and psychological well-being. Three questionnaires were administered to measure levels of affective instability, the ability to manage fluctuating affective states, and overall psychological well-being. The findings suggest that (1) individuals with diagnoses experience affective lability and difficulty regulating emotions at a greater rate than those without, (2) higher affective lability scores are consistent with more significant emotion dysregulation and lower overall psychological well-being, and (3) scores on the Affective lability Scale (ALS) and the Difficulties in Emotional Regulation Scale (DERS) are reliable predictors of one’s estimated Global Assessment of Functioning (GAF) scores. Although causation has not been established, the evidence suggests that individuals with diagnoses experience greater difficulty in regulating their emotions, have greater affective lability, and experience diminished psychological well-being and day-to-day functionality. Certain anecdotal evidence suggests that emotional lability can be endogenous and affect multiple aspects of an individual’s social, occupational, and personal life. By revising the existing literature and the present findings, the authors provide insights into the significance of endogenous factors in the context of affective lability and offer suggestions for future research.

## 1. Introduction

Previous research has investigated the multidimensional nature of emotion regulation and affective stability, particularly among individuals diagnosed with personality and mood disorders. Affective instability, the propensity to experience intense and fluctuating emotional and affective states, has been associated with poor well-being, disrupted clinical functioning, and maladaptive social behaviors [1,2]. In much of the existing literature, the terms affective instability and affective lability are often confused and used interchangeably [3]. Renaud and Zacchia [3] clarify and define the term as an inherited temperamental trait involving affective valence, affective amplitude, a low reactivity threshold to environmental stimuli, dysregulation, and rapid and unpredictable shifts in affect. A key aspect of affective instability, affective lability, refers to the excessive and unpredictable deviations from one’s emotional baseline [1]. Such patterns can lead to distress and numerous psychological issues [4]. Affective instability has been described in the context of various disorders, such as borderline personality disorder (BPD) and complex post-traumatic stress disorder (C-PTSD) [5]. Since the International Classification of Disease (ICD-11) diagnostic criteria were updated in 2018, C-PTSD has been used to describe cases in which an individual meets all the criteria for PTSD but exhibits additional disturbances in self-organization (DSO): negative self-concept, interpersonal disturbances, and affect dysregulation. Further emphasizing the significant role affective dysregulation plays within the development and expression of the disorder, the DSM-5 added multiple additional symptoms to its description of PTSD, which included mood changes [6]. Here, we aim to provide a comprehensive illustration of emotional instability, its components, significance, and its connection to other psychological concepts. Due to common confusion, it is necessary to clarify what emotional instability is and what it is not. 

### 1.1. Neuroticism

Affective instability and affective lability should not be confused with neuroticism, the tendency for an individual to experience negative affect (NA), such as sadness, shame, and anger [7]. As stated, affective lability and instability refer to the characteristics of emotional responses, while neuroticism refers to an individual’s predisposition to experience negative emotionality [7]. Through statistical analysis, these are related yet distinct constructs, each with different correlates. For example, Miller and Pilkonis [7] found that high neuroticism was associated with internalizing emotional experiences, while affective instability was associated with more impulsive emotional expressions. Both have been related to psychological disorders, such as borderline personality disorder (BPD) [7]. However, some research has suggested that affective instability maybe even more important for the development of psychological disorders and provides unique value when predicting functionality, even when neuroticism is controlled [8].

### 1.2. Emotional Regulation

#### 1.2.1. Conceptual Frameworks

Associated with affective instability is the idea that part of psychological well-being emanates from one’s ability to manage these emotions [9], otherwise known as emotion regulation. Within this study, “emotional regulation” refers to one’s ability to intrinsically moderate emotions and emotional responses [10]. This process can apply to negative or positive emotions and refers to one’s ability to increase or decrease prominence. It is most commonly observed that adults tend to downregulate negative emotions (e.g., sadness, anger, frustration) [10]. With optimal capacity to regulate, individuals can modify their emotions, monitor when they arise, and moderate how they convey them [11]. One’s emotional regulation is associated with greater scores of well-being, better relationships, and greater academic and vocational success [12]. Numerous theoretical frameworks seek to identify and describe differences among individual’s emotional regulation capabilities. Historically, there has been a lack of agreement on these conceptual frameworks and validated measures that capture all the complexities of emotional regulation [13]. For example, some conceptualizations emphasized controlling and reducing emotional expression (predominantly negative emotionality). In contrast, others stressed the importance, function, and utility of specific emotions (often dependent on the context of a situation). In support of a multidimensional framework, Gratz and Roemer [13] provide an integrative and comprehensive model of emotional regulation based on the idea that intact emotional regulation involves (a) emotional awareness and understanding, (b) acceptance of emotions, (c) control of impulsive actions and the ability to engage in goal-directed behavior when experiencing negative affect, and (d) the ability to use situationally appropriate emotion regulation methods to meet the demands of a situation [13]. While there are several competing models, there is a shared emphasis on one’s ability to regulate one’s emotions in ways that are optimal for functioning within the present environment

#### 1.2.2. Implications

While affective instability is a potential risk factor for emerging personality and mood disorders (e.g., bipolar disorder), the ability to regulate emotions is believed to counteract the negative implications of experiencing severe and variable emotions. Affective processing and regulation regarding mood psychopathology may implicate predictive assessments that can alter treatment interventions and the overall course of various disorders [4,14]. Emotions are often considered to be responses to external stimuli. However, the growing literature suggests that some affective phenomena can be elicited by endogenous factors (e.g., hormonal imbalance, mental imagery, memories of past events, etc.) [14,15,16]. Sometimes, these emotional episodes arise abruptly and from seemingly unidentifiable causes. Interestingly, these endogenous affective responses physiologically and subjectively resemble responses to external stimuli and exogenous disturbances and may indicate psychopathology. More recent research has suggested that these endogenous patterns can be “trained”, possibly through therapeutic methods, to increase the frequency of positive emotions, even in the presence of external stressors [15]. This phenomenon appears to be innately related to affective lability. A better understanding of affective lability may be important for understanding how emotions arise from seemingly “nowhere” and could provide insight into how such endogenous responses can be managed. Emotional regulation appears to have a vital impact on an individual’s well-being, possibly by mitigating the adverse effects of emotional instability/lability.

### 1.3. Psychological Well-Being

Without emotional regulation resources, affective instability and lability have a negative impact on an individual’s psychological well-being (PWB) [17]. PWB is a complex construct defined by the World Health Organization as a state of mind in which individuals can work productively, reach their full potential, and properly manage daily stressors [18]. While continuously debated, the proposed key components of PWB include positive emotions, autonomy, personal growth, positive relationships, and life satisfaction. It indicates good mental and physical health and a higher life expectancy [18].

In the present study, we aim to expand upon existing empirical research by investigating the prevalence of affective lability, a key component of affective instability, and the capacity to self-regulate emotions among college students. Previous research has primarily focused on the relationship between affective instability and lability, as well as emotion regulation, within personality and mood disorders or has placed significant emphasis on its relationship with children [19,20]. The comprehensive goal of the current study is to understand how affective lability may develop and affect college students’ psychological well-being, regardless of whether they have been diagnosed with a psychological disorder, and to examine how this corresponds to their ability to regulate their emotions. We hypothesize that (1) higher affective lability scores will be associated with greater difficulty in regulating emotions and lower psychological well-being, (2) individuals with diagnoses will experience higher levels of affective lability and difficulty in regulating emotions compared to those without diagnoses, and (3) scores from the Affective Lability Scale-18 (ALS–18) and the Difficulties in Emotion Regulation Scale-Short Form (DERS–SF) will be significantly related to an individual’s Global Assessment of Functioning.

## 2. Materials and Methods

### 2.1. Participants

A total of 420 students from a Midwest university participated in the study. Online recruitment was conducted through the Study Scheduling System (SONA), and participants were offered compensation in the form of class credit. Eligibility criteria required participants to be aged eighteen years or older and not residing in the European Union at the time of study completion. Informed consent was obtained, and participation was entirely voluntary. Participants were free to withdraw from the study at any point, and in such cases, their responses were excluded from the data.

Additionally, those who provided incomplete or inconsistent responses throughout the survey (e.g., reporting an age of onset for affective lability symptomatology that was not possible based on their recorded current age, such as reporting an age of onset as 40 while their current age was 19) were excluded. These participant’s lack of consistency suggested a lack of engagement in providing quality responses to the survey, thereby compromising response integrity. Furthermore, participants who did not provide consent, indicated residency in the European Union, had unfinished responses, or displayed inconsistency were also excluded from the dataset. As a result, a total of 328 responses were used for analysis. We used G*Power (version 3.1.9.7) to calculate the a priori power analysis. The estimated minimum sample size was 111. We anticipated difficulties in increasing the sample size of the diagnosed group, so we increased the overall sample to improve the results. Kim and Park (2019, p. 334) [21] state, “Relatively larger sample sizes are needed in such cases to obtain similar statistical results”.

Participants’ ages ranged from 18 to 35, with a mean age of 19.0 (SD = 2.08) years. Seventy-four percent of participants identified as female. The racial/ethnic composition of our sample was reported as 78.3% White, 8.9% Hispanic or Latino, 5.3% African American or Black, 3.4% Asian or Pacific Islander, 3.1% Multi-Ethnic, less than 1% reported as American Indian or Alaskan Native, and less than 1% did not report their racial/ethnic identity. Participants were asked to indicate whether they had previously been diagnosed with a psychological or medical disorder/illness that may influence their responses and to specify the diagnosis in a free-response blank.

#### Data Analysis

The questionnaire results were organized and analyzed using SAS software, Version 9.4 of the SAS System for Unix [22]. Based on our study design, we checked for the possibility of variable inflation and/or multicollinearity (VIF). We used SAS to evaluate the degree of VIF in our data. This estimated VIF to be 1.67. This did not exceed the commonly accepted threshold of 4.0 and suggested that VIF was not influencing our findings. Regression analysis was performed to control for shared variance in our predictor variables and should be interpreted in such a manner. Based on the Folded F test, measures of skewness between −0.5 and 0.5, and kurtosis < 1, the data evidence adequate normality. All 14 of 15 comparisons remain significant using the Benjamini and Hochberg correction. Applying the more stringent Bonferroni correction for 15 comparisons results in a (0.05/15) *p* < 0.0033 for significance. All 14 comparisons remain significant.

### 2.2. Measures

#### 2.2.1. The Short Affective Lability Scale (ALS–18)

The short form of the Affective Lability Scale (ALS–18) is adapted from the Affective Lability Scale (ALS) and is designed to measure one’s tendency to experience fluctuations in mood [23,24]. These quick mood shifts may range from a baseline (“normal”) mood to anger, anxiety, depression, and elation. Additionally, the scale measures the propensity to change between anxiety and depression, along with depression and elation [25]. Prompts on the scale range from: “I shift back and forth from feeling perfectly calm to feeling uptight and nervous” and “Sometimes I feel extremely energetic one minute, and then the next minute I might have so little energy that I can barely do a thing”; to “I frequently switch from being able to control my temper very well to not being able to control it very well at all”. Participants rate the items on a 4-point scale, from 1 = *Very undescriptive* to 4 = *Very descriptive*. As the original scale is extensive, its shortened form was used instead of the full 54-item questionnaire. Due to its high convergent validity with the original scale and high construct validity, we concluded that the ALS–18 would be an excellent measure of affective lability for the present study [25].

#### 2.2.2. Difficulties in Emotion Regulation Scale—Short Form (DERS–SF)

The short form of the Difficulties in Emotion Regulation Scale (DERS–SF) is adapted from the full-length original Difficulties in Emotion Regulation Scale (DERS), which measures dysregulation in terms of six dimensions: (a) lack of awareness of emotional responses; (b) lack of emotional response clarity; (c) nonacceptance of emotional responses; (d) restricted use of effective emotional regulation strategies; (e) difficulty controlling impulsive behavior when experiencing negative affect; and (f) and limited ability to carry out goal-oriented behavior while experiencing negative emotions [13,26]. The DERS is widely used as a valid and reliable measure of emotional regulation for clinical and research purposes. Example prompts include: “When I’m upset, I acknowledge my emotions”, “When I’m upset, I become out of control”, and “When I’m upset, it takes me a long time to feel better”. Participants rate the items on a 5-point scale, ranging from 1 = *Almost never* to 5 = *Almost always*. The DERS demonstrates high levels of internal consistency (α = 0.93) and good reliability (ρ = 0.88, *p* < 0.01). Acknowledging the need for a shorter version, the DERS–SF was utilized in the present study. The DERS–SF maintains the psychometric properties in the original scale, with a covariance of 81–96% [24].

#### 2.2.3. Ryff’s Psychological Well-Being Scale (PWS–18)

**PWS–18.** The short form of the Psychological Wellbeing Scale (PWS–18), adapted from the original Psychological Wellbeing Scale (PWS), is designed to assess overall well-being across six dimensions of wellness: autonomy, environmental mastery, personal growth, purpose in life, positive relations with others, and self-acceptance [18,27,28]. This six-factor theoretical model has been found to reliably measure positive functioning and well-being [27]. Example prompts from the scale include: “For me, life has been a continuous process of learning, changing, and growth”, “I have not experienced many warm and trusting relationships with others”, and “The demands of everyday life often get me down”. Participants rate the items on a 7-point scale, ranging from 1 = *Strongly agree* to 7 = *Strongly disagree*. Due to its length, a preliminary study by Ryff and Keyes [27] created a shortened version of the PWS consisting of 18 items. Scale internal consistency ranged from 0.33 (Purpose in Life) to 0.56 (Positive Relations With Others) [28]. Ryff and Keyes [28] maintain that the low to moderate alpha coefficients were attributed to the small number of indicators per subscale, which had been chosen to maximize conceptual breadth.

#### 2.2.4. Global Assessment of Functioning (GAF)

**GAF.** Closely related to this conceptualization of PWB is the Global Assessment of Functioning (GAF). The GAF is a clinical measure of general psychosocial disability determined by reviewing biopsychosocial factors in an individual’s life. It is synonymous with Axis V of the Diagnostic and Statistical Manual of Mental Disorders, Fourth Edition Text Revision (DSM-IV-TR) [29]. This measure has been shown to be valid and reliable [30]. This continuous scale, ranging from 1–100 (1 denotes extreme psychosocial impairment and 100 indicates no symptoms and superior functioning in a wide range of activities; optimal psychosocial well-being), assesses severity, impairment, and overall quality of one’s life and mental health.

### 2.3. Procedure

The entirety of the study was conducted and administered using the online survey platform Qualtrics XM. Participants completed the study on their own devices and on their own time. Before completing the questionnaires, each individual indicated whether they met the participation criteria, followed by providing informed consent. At this point, participants were asked to provide demographic information (i.e., age, race). The *ALS–18* was then administered, followed by a series of questions regarding whether the participant has a diagnosis that they believe affects their answers to the scale.

Additionally, participants indicated to what extent they feel the experiences described in the *ALS–18* affect their day-to-day lives in a multiple choice Likert scale. Participants also specified the ages at which they first began to experience symptoms and received a diagnosis. The DERS–SF and PWS–18 were then administered, respectively. Upon completing the questions and scales, participants were thanked for their contribution and debriefed about the study.

### 2.4. Analysis

To better understand the impact of affective lability and difficulties regulating emotions on participants, two researchers independently analyzed the data using the Global Assessment of Functioning (GAF) Scale and responses to various questions. This included a specific open-ended question as a way of determining a GAF score for each participant. The three related questions under review included: *To what extent do you believe the previously described experiences affect your life? Have you ever been diagnosed with a medical or psychological illness that you believe would affect your answers to the questions directly above? To what extent do you believe the previously described experiences affect your life, and in what way?* The researchers reviewed the GAF scoring criteria, initially independently reviewed and rated, and then compared conclusions. The kappa was above 95%, and where there was divergence, the two discussed and then came to a consensus for each participant’s estimated GAF score.

## 3. Results

### 3.1. Participants and Diagnoses

The results of this study show that 32.9% of the participants (*n* = 108) reported having been diagnosed with a medical and psychological condition (e.g., Depression, Anxiety, Bipolar Disorder, ADHD, OCD, Ehlers-Danlos Syndrome, etc.) that they believed affected their answers to the survey.

### 3.2. Independent Samples t-Test

**Figure 1:**
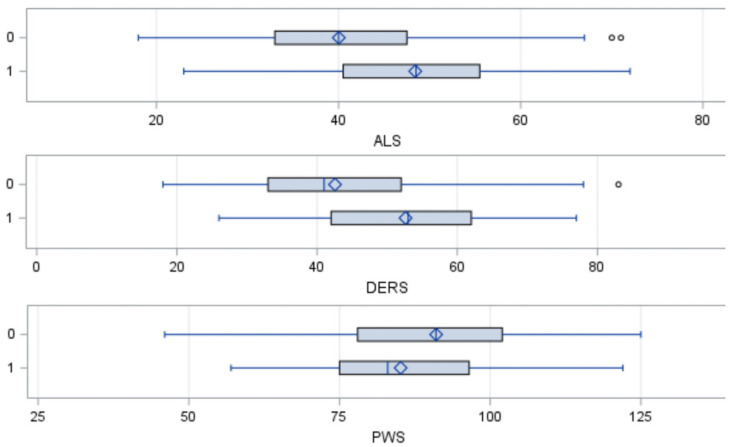
Independent *t*-tests Box Plots. Note. This figure depicts the results of the independent *t*-tests. ALS = Total Affective Lability Scale Score, DERS = Total Difficulties in Emotion Regulation Scale Score.

After dividing the sample into two groups based on a self-reported psychological or medical diagnosis (No Diagnosis vs. Diagnosis), the results revealed significant differences in ALS–18 scores, DERS–SF scores, and PWS–18 scores between groups (*p* < 0.0001). On average, participants with a diagnosis (*M* = 48.42, *SD* = 11.18) reported higher ALS–18 scores compared to those who did not have a diagnosis (*M* = 40.02, *SD* = 11.04), *t*(326) = −6.44, *p* < 0.0001. As expected, participants with a diagnosis (*M* = 85.14, *SD* = 15.33) had lower PWS–18 scores than those without a diagnosis (*M* = 91.03, *SD* = 15.25), *t*(326) = 3.28, *p* < 0.0001. Table 1 presents the differences between the two groups on the ALS–18, DERS–SF, and PWS–18 inventories. Figure 1 presents the results of the independent t-tests.

### 3.3. Correlational Findings

Correlational analysis showed a strong positive correlation between ALS–18 and DERS–SF scores, *r*(328) = 0.64, *p* < 0.0001. Additionally, moderate negative correlations were found between ALS–18 and PWS–18 scores, *r*(328) = −0.45, *p* < 0.0001, as well as between DERS–SF and PWS–18 scores, *r*(328) = −0.60, *p* < 0.0001. A significant negative correlation was also observed between the reported age of experiencing affective lability symptomatology and the estimated Axis V score, *r*(87) = −0.22, *p* = 0.04.

Furthermore, there were moderate positive correlations between diagnostic impact and ALS–18 scores, *r*(327) = 0.58, *p* < 0.0001, as well as with diagnostic impact and DERS–SF scores, *r*(327) = 0.53, *p* < 0.0001. Table 2 provides the Pearson correlation coefficients for the study variables.

### 3.4. Regression Analysis

As depicted by Table 3, regression analysis was conducted to examine the effects of participants’ total ALS–18 and DERS–SF scores on their designated Axis V score. The overall regression was statistically significant (*R*^2^ = 0.30, *F*(2, 325) = 69.33, *p* < 0.0001). ALS–18 and DERS–SF scores predicted one’s estimated Axis V score when entered simultaneously (ꞵ = 111.35, *p* < 0.0001). Such analysis was not conducted for psychological well-being because the previously established correlations were negative. 

## 4. Discussion

Previous research has studied the multidimensional determinants of affective instability, lability, and difficulties with emotion regulation, specifically in congruence with personality and mood disorders. While affective lability is a potential risk factor for developing said disorders, one’s ability to effectively regulate emotions is thought to counteract these intense and fluctuating moods. Our results are consistent with prior findings of higher self-reported affective instability/lability scores associated with greater difficulty regulating emotions and lower self-reported psychological well-being. In addition, our findings are consistent with observations of affective lability and difficulties in emotion regulation being closely related to the diagnosis of a psychological disorder. Since both were simultaneously associated with having a diagnosis, this may further validate the idea that these phenomena can be predictors of emerging psychopathologies [31]. Similarly, our findings supported our hypothesis that the total ALS–18 and DERS–SF scores would be positively correlated and be good predictors of overall psychological well-being and a designated Axis V score.

### 4.1. Affective Lability and Emotion Regulation on Psychopathology

As stated and in line with prior work, our findings suggest that those reporting higher affective lability scores are more likely to experience difficulty regulating their emotions and report more significant struggles with overall psychological well-being and general functioning (Axis V). In addition, those who reported higher affective lability were also more likely to indicate being diagnosed with a psychological or medical disorder/illness. As noted, these most commonly include major depressive disorder (MDD), generalized anxiety disorder (GAD), obsessive-compulsive disorder (OCD), bipolar disorder, attention-deficit/hyperactivity disorder (ADHD), and posttraumatic stress disorder (PTSD).

### 4.2. Affective Lability and Axis V

Our findings suggest that affective lability may impair psychological well-being and overall general functioning. Using the Global Assessment of Functioning (GAF) Scale, estimated Axis V scores provided insight into the impact of affective lability on one’s mental health and overall quality of life. While our design could not conclusively show that affective lability was responsible for poorer psychosocial well-being, the correlations do suggest that affective lability plays a role in the detriment of one’s mental health and functioning. In addition, our use of Axis V criteria provides an overarching idea of one’s illness severity and offers promise in evaluating treatment and outcomes across all diagnoses [32].

When participants were asked to provide insight into how they believed affective lability and difficulties with emotion regulation had affected their lives, common themes emerged. Problems with school, work, and social relationships (e.g., family and friends) were commonly reported. Anecdotally, one participant indicated, “I am incapable of doing things alone, get super nervous in social situations, tend to lean on people for things, and will suddenly feel super sad out of nowhere”. Lack of motivation and fatigue were also common themes presented in these responses. One participant disclosed that they find themselves “lacking motivation or energy to do pretty much anything”, while another revealed their struggle with the inconsistency of their emotions, saying, “When I am high energy, I become extremely sociable, productive, and am overall in a great mood. But when I am low energy, I feel like I am in a fog, frozen in one place. It makes me stressed because even when I have things to do, I no longer have the capacity to do them”.

### 4.3. Affective Lability’s Emergence and Duration

There is little to no research on affective lability outside of being a symptom of numerous psychological disorders. While it is thought to put one at risk for developing a disorder, there is no definitive work on why or how it emerges. There is a well-developed understanding of how external factors can play a role in influencing affective states, but a lack of knowledge regarding potential endogenous factors, such as the role of the cerebellum or hormonal abnormalities [33,34]. As stated, endogenous emotional instability often presents as altered emotional states that appear to have no causal link to any environmental change/stimuli. Emotionality or affective lability changes may occur as a response to a recalled memory or one’s stream of thought [15]. Previous researchers have claimed that these emotional responses are elicited by interacting with different “modalities” (e.g., visual imagery, semantic processing, etc.). Visual imagery, which can have psychological effects similar to actual perception, maybe a particularly important contributor. [15]. However, it is likely that these different modalities interact when eliciting such emotional responses [15]. This description shares similarities with one anecdotal comment given by a participant: “I will suddenly feel super sad out of nowhere”. Such anecdotal reports suggest that affective lability may include a stand-alone subtype variant. They may consist of brief intervals lasting approximately 15–20 min (absent any evident cues or causes that occur several times a week) and can be quite perplexing and disquieting. We hope our study might prompt future work focused on a brief episodic endogenous type of affective instability and any concomitant cognitive or behavioral sequelae.

Lastly, our findings suggested that an individual’s estimated Axis V score was associated with an earlier age of emerging affective lability symptomatology. More research is needed to establish whether there is a relationship between endogenous etiology and age of onset. If this is the case, it may be important when devising treatment plans for clients experiencing endogenous emotional instability. Treatment plans may have to incorporate “endogenous emotion generation training”, as described by Engen et al. [15].

### 4.4. Limitations

What our findings were unable to conclude was the role affective lability plays in a psychological disorder. Does this phenomenon put one at risk and be able to predict the onset of a disorder, as Taylor et al. [14] theorize with specifically bipolar disorder, or does it elicit from the disorder as a symptom? Understanding the role of affective lability in psychological disorders can enhance diagnostic certainty and reduce the risk of psychopathological development through early intervention [14].

The current study may also be limited by the methodology used. The current study utilized self-report measures, potentially more subjective than alternative methods, such as ecological momentary assessment (EMA). EMA is a useful method for measuring such phenomena in real-time. For example, Miller et al. [35] measured participants’ emotional states and their fluctuation patterns by providing each individual with a Palm-Pilot, on which they answered questions about their emotions at certain scheduled times of the day. This is useful for preventing possible recall bias and obtaining more accurate time stamps. By collecting data in real-time using EMA, the participant no longer needs to rely on their memory, which could lead them to submit inaccurate information. However, while self-report methods may be more susceptible to recall bias and may not collect as precise time measurements, relevant findings by Miller et al. [35], based on EMA, have been consistent with previous findings using self-report questionnaires. In other words, the results and/or conclusions from some studies using EMA have been consistent with results from self-report methods, suggesting the difference in accuracy may not be a significant problem. However, EMA risks being perceived as an annoyance by participants and may be prone to missing data due to noncompliance [36]. Because both methods have advantages and disadvantages, future research should replicate the current study using EMA to compare findings.

Because those experiencing heightened affective lability appear to lack the capacity for emotion regulation, increasing this capacity may be a crucial component of treatment [3,14]. More research is needed to understand the direction of this relationship. Our findings show a moderately strong correlation between affective lability and difficulty regulating emotions but do not determine how they may influence each other. Does one’s inability to regulate emotions precede and worsen the oscillation and intensity of varying moods, or might these conditions be inversely related?

### 4.5. Future Research

Several unanswered questions remain regarding affective lability and its relation with other psychological phenomena, how it emerges, how frequently these mood shifts occur, and for how long. Nevertheless, a better understanding of affective lability may prove helpful in predicting the onset of psychopathologies and implementing intervention strategies related to emotion regulation. Future research should further investigate potential factors that mediate or moderate an individual’s ability to manage their emotional states. Future research should also explore the relationship between endogenous affective lability and age of onset and how an endogenous etiology influences the utility of current treatment approaches.

## 5. Conclusions

As we have seen, individuals diagnosed with a psychological disorder experience affective lability at a higher rate than those without a diagnosis and have greater difficulty regulating their emotions. Such difficulties are associated with diminished psychological well-being and can hinder their day-to-day functioning. Anecdotal evidence suggests that affective lability impacts various aspects of individuals’ lives, including work, school, and relationships, with some reporting diminished productivity and a reliance on others. Although the causes of affective lability are not fully understood, we emphasize the potential for some instances to be endogenous. We hope that this study will stimulate further research on affective instability, its associated risks, development, interventions, and the significance of endogenous factors. We propose that these insights into affective lability can be applied in clinical practice when working with clients who report similar experiences. These insights could assist clinicians in gaining a better understanding of their client’s concerns, and how to serve them. Armed with this information, clinicians may be able to provide clarity to clients who may feel confused or frustrated by their seemingly unpredictable emotional experiences.

## Figures and Tables

**Table 1:** Differences between participants without a psychological/medical diagnosis versus participants with a psychological/medical diagnosis on the ALS–18, DERS–SF, and PWS–18 inventories.

No Diagnosis	Diagnosis	Mean (SD)	df	*t*	*p*	Cohen’s d
ALS–18	40.02 (11.04)	48.42 (11.18)	326	−6.44	<0.0001	0.76
DERS–SF	42.55 (12.36)	52.62 (12.88)	326	−6.84	<0.0001	0.80
PWS–18	91.03 (15.25)	85.14 (15.33)	326	3.28	0.0011	0.39

Note: N = 328; ALS = Total Affective Lability Scale Score, DERS = Total Difficulties in Emotion Regulation Scale Score, PWS = Total Psychological Well-Being Scale Score.

**Table 2:** Correlation Matrix of the Study Variables.

Variable	M (SD)	1	2	3	4	5	6	7	8
1. Axis V	84.78 (0.50)								
2. Diagnostic	1.26 (0.94)	−0.86 **	—						
**Impact**									
3. Emotion	1.23 (0.75)	−0.34 **	0.35 **	—					
**Presentation**									
4. ALS	42.79 (11.75)	−0.52 **	0.58 **	0.28 **	(0.92)				
5. DERS	45.87 (13.38)	−0.46 **	0.53 **	0.19 *	0.64 **	(0.90)			
6. PWS	89.09 (15.50)	−0.32 **	−0.33 **	−0.09	−0.45 **	−0.60 **	(0.85)		
7. Age	18.94 (1.80)	0.01	−0.04	−0.01	−0.04	0.04	−0.05	—	
8. Gender	0.73 (0.44)	0.01	0.01	0.00	0.03	0.06	−0.02	−0.04	—

Note: N = 328; Cronbach’s Alpha values are on the diagonal, ALS = Total Affective Lability Scale Score, DERS = Total Difficulties in Emotion Regulation Scale Score, PWS = Total Psychological Well-Being Scale Score, Gender is coded as follows: male = 0, female/non-binary = 1. * *p* < 0.001, ** *p* < 0.0001.

**Table 3:** Regression Analysis.

Parameter Estimates								
Variable	DF	B	Standard Error	*t* Value	Pr > |*t*|	β	Variance Inflation	95% Confidence Limits
Intercept	1	111.3	2.3	47.75	<0.0001	0	0	[106.8, 115.9]
ALS	1	−0.39	0.06	−6.26	<0.0001	−0.38	1.68	[−0.52, −0.27]
DERS	1	−0.21	0.06	−3.72	0.0002	−0.22	1.68	[−0.32, −0.10]

Note: N = 328; ALS = Total Affective Lability Scale Score, DERS = Total Difficulties in Emotion Regulation Scale Score.

## Data Availability

The raw data supporting the conclusions of this article will be made available by the authors on request.
